# The Atlanta Medical Society

**Published:** 1883-03

**Authors:** 


### Societies.

### THE ATLANTA MEDICAL SOCIETY.

*Reported for the Atlanta Medical Register.*

ATLANTA, February, 1883.

The President, Dr. W. S. Armstrong, in the chair.

Dr. G. G. Roy reported the following case: Mrs. L., aged nineteen years, the mother of a healthy child seven months old, contracted intermitent fever at Augusta, in the month of August last. He was called to see her about three months ago, after she had been attended by several physicians, and found her suffering from intestinal obstruction, apparently intussusception of the upper bowel. She had had no action for a fortnight, high fever and constant vomiting, which the nurses described as stercoraceous. He observed that she apparently experienced great difficulty in swallowing, which she attempted to aid by pressure with the hand placed upon the left side over the cardiac orifice of the stomach, at which point, she said, her food seemed to lodge. Pressure helped her to get it down. She was very anæmic, skin of an icteric hue, excessively irritable stomach and enlarged spleen. Constipation had lasted for two weeks, and was not relieved for a week longer. He ordered twenty grains of calomel, repeated in two hours. This dose produced no effect upon the bowels but caused severe salivation. It was followed by two drachms each of sulphur and cream of tartar which brought about free alvine discharges followed by relief of the dysphagia. This was the second attack she had experienced and of the first attack, about five years ago, she gave the following history: In 1878, after a romp with some children in a very warm room, she seated herself by a stove and soon became very drowsy; this was promptly followed by nausea and vomiting of a bloody matter. Blood continued to ooze from her mouth, unaccompanied by cough, for several hours. She was, at that time, very plethoric. The trouble in the œsophagus, just described, then, for the first time, made its appearance and for two years she was unable to ingest solid food and was sustained by liquid diet exclusively. As the result of a course of treatment which she underwent in Augusta, where she then resided, she obtained relief, and remained well until the occurrence above alluded to.

Dr. W. S. Elkin reported the case of an old man, a physician from Kentucky, who had suffered for years from Bright's disease of the kidneys. The urine contained a slight trace of albumen but no casts. No valvular lesions of the heart, but hydro-pericardium and the legs were anasarcous. He was an opium eater, and his arms showed numerous marks of hypodermic injections. Digitalis and squills acted well for a few days, these remedies then failed and pilocarpin—one-third of a grain of the nitrate—caused copious sweat and diuresis—after a few days the digitalis was resumed, and these two agents were subsequently alternated. The patient died very suddenly a few days ago. After a nap of three-quarters of an hour, he awoke complaining of chilly sensations, and almost immediately fell to the floor and instantly expired.

Dr. W. D. Bizzell said he had used pilocarpin and fluid extract of jaborandi in cases of Bright's disease but had never discovered an real benefit from the remedy, but on the contrary had observed some positively dangerous effects. Alarming prostration had followed the adminis·tration of one-quarter of a grain of pilocarpin and of one drachm of fluid extract of jaborandi.

Dr. James B. Baird, upon call, reported a case of typhoid fever recently treated with successful issue, which presented two rather rare and decidedly dangerous complications.

A young gentleman, aged sixteen years, developed a well-marked case of typhoid fever last fall, soon after his return to the city from a mountainous summer resort. The case was first seen on the fifth day of the fever. It then presented no features of special note. The eruption, in due time, was conspicuous, unusually so, referable, in part, no doubt, to the exceeding fairness of the skin, which, in its whiteness, resembled that of a very blonde girl. The abdominal symptoms were notably prominent. The nervous symptoms were not so pronounced as usual, though there was some mental dullness, carphologia, subsultus, etc. Temperature ranged from 102° F., in the morning, to 103½° F. in the evening; pulse about 120. On the fourteenth day from the onset of the fever, without warning, two copious hemorrhages from the bowels occurred. The prostration resulting was extreme. The patient was blanched, lips white and he was almost pulseless. Whisky, opium and acetate of lead were freely administered. Diet as before was exclusively fluid. His bowels were confined for ten days, after which warm water enemata, terminated by a few bulbfulls of sweet oil, were administered with satisfactory results. No blood staining the semi-solid stools. At the end of four weeks he was entirely free from fever. His symptoms were all favorable and convalescence was declared established. After three days of complete immunity his fever reappeared. Evening temperature 103½° F. Extreme abdominal tenderness recurred, and, without assignable cause, he developed and passed through a second attack, which lasted about three weeks. This case presented the only distinct relapse he had ever met with in this disease. He has seen three cases of intestinal hemorrhage, two recovered and one died; though the fatal issue was not directly dependent upon, and did not seem to be influenced by the hemorrhage as it did not ensue for several weeks after it occured, and there was no recurrence of the bleeding.

Dr. W. S. Armstrong said he thought the history of this case was very interesting, especially the features relating to the relapse. It called to mind some of his own experience in this disease. About three years ago he treated a case which suffered a relapse—the second instance in his practice—in the attack nothing remarkable or unusual occurred. The boy was up and about the house for several days when he was again prostrated and passed through a second attack similar in every respect to the first. The following summer two brothers of this boy, living at home, had typhoid fever; both recovered, and strange to relate, last summer--one year later—both of these boys had a second attack.

The whole series of events showed remarkable coincidences. He has seen but little of intestinal hemorrhage in this disease—only three cases all told—two of these died. He thinks the gravity of the complication depends upon the suddenness and amount of the hemorrhage, the condition of the patient and attendant circumstances.

Dr. Armstrong, continuing, reported progress in the case of blood poisoning in a police officer as the result of a stab by a prisoner, previously detailed [and published in the January number of the REGISTER.] It will be remembered that at the date of the last report he was walking and riding out, and his assailant, in view of his great improvement, had been released on bond.

On January 7th, he complained of pain in the calf of the left leg—which was tender and swelled. He had phlebitis limited to the external saphena. Pneumonia of the left lung supervened but was not severe, and by January 27th, he was sitting up. In a day or two he was called to see him and found a general phlebitis of the left leg; pulse, 120; general disturbance, great; the whole leg tender, which extended even above Pouparts ligament. The trouble has gradually subsided, and a few days ago he was again sitting up The case has been a most remarkable one. It is now five months since the receipt of the wound—the prime cause of all his sufferings.

Apropos of blood poisoning, he related the following history: Five weeks ago he delivered a lady of a mature child. All went well for five or six days, about which time the left mammary gland became intensely inflamed and enormously swelled. There were no lumps or cakes. but it was erysipelatous, the skin having a hard feel like exudation had occurred in the subcutaneous areolar tissue. She was soon alarmingly ill. Temperature, 104° F. Some delirium—uterus tender to external manipulation, no tympanitis, no peritonitis or pelvic cellulitis. He was impressed with the idea that he had a case of septicæmia —blood poisoning. The treatment consisted in the exhibition of opium in large quantities, veratrum, vaginal injections of a two per cent. solution of carbolic acid, and supporting diet. In about ten days the symptoms yielded.

But about eight days ago was called to find the external and internal saphenous veins inflamed. This occurrence confirmed his opinion of the case. There was no very great general disturbance. That disorder has about subsided. She is now nourishing her child, eating enough and doing well.

